# Decaying diffusion-based similarity measure of nodes and multi-scale modular structure finding in brain networks

**DOI:** 10.1186/1471-2202-12-S1-P171

**Published:** 2011-07-18

**Authors:** Tae-Wook Ko, Eun-Youn Kim

**Affiliations:** 1 Computational Neuroscience Team, National Institute for Mathematical Sciences, Daejeon, 305811, Republic of Korea

## 

Information about brain structure, especially information about how neurons or brain regions are wired is essential for the study of brain function and brain disease [1,2]. Recently, brain networks have drawn increasing attention due to the success of graph-theoretic or statistical physics-based network analysis of complex systems such as WWW, social networks, and metabolic networks [3] and the development of techniques for obtaining brain connectivity information. Graph theory enables us to describe a neural system simply as a graph which is a collection of nodes corresponding to neurons, neuronal population, or brain regions and links corresponding to the structural links or statistical or causal relationships between the nodes [1,2]. Since elements of the brain group together to perform functions, it is important to find out how closely two elements are related and which elements form a module which can be defined as a subsystem of a system that is functionally or structurally distinct from other parts of the system. Here, we introduce a similarity measure between nodes based on random walks in which the diffusion flux decays with the path length in the network [4]. The similarity is measured as how similarly two diffusion processes starting from two different nodes affect other nodes of the network. The decaying diffusion process reveals the landscape of the networks and using this similarity measure, we propose a multi-scale clustering method to uncover hierarchically modular structures in brain networks. In hierarchically modular networks (HMN), modules consist of sub-modules and those sub-modules may be composed of smaller sub-modules and so on. HMNs reflect the functional segregation of local areas and global integration in complex systems such as the brain and society. We incorporate multi-scale multiple grouping which gives the information about the frequency of nodes being grouped together in various sizes of groups. Then, we sequentially remove weak ties, which are defined by low co-membership frequency, and find out connected components which correspond to the modules of the system. We apply our method to analyze hierarchical modular structure of macaque monkey brain network and discuss the results. This method can be used to analyze both structural and functional brain networks.
